# Correction: Origin and evolution of transporter substrate specificity within the NPF family

**DOI:** 10.7554/eLife.46989

**Published:** 2019-04-02

**Authors:** Morten Egevang Jørgensen, Deyang Xu, Christoph Crocoll, Heidi Asschenfeldt Ernst, David Ramírez, Mohammed Saddik Motawia, Carl Erik Olsen, Osman Mirza, Hussam Hassan Nour-Eldin, Barbara Ann Halkier

Jørgensen ME, Xu D, Crocoll C, Ernst HA, Ramírez D, Motawia MS, Olsen CE, Mirza O, Nour-Eldin HH, Halkier BA. 2017. Origin and evolution of transporter substrate specificity within the NPF family. *eLife* **6**:e19466. doi: 10.7554/eLife.19466.

Published 3, March 2017

In our revision, we constructed large multiple sequence alignments and built 3D-homology models to put identified transporter substrate specificities into structural contexts. David Ramirez (DR), Heidi Asschenfeldt Ernst (HAE) and Prof Osman Mirza (OM) performed this work. DR was physically located together with the corresponding authors and he made figures Figure 8, Figure 8–figure supplement 1 and Table 1 in collaboration with and based on data generated by HAE, OM, and DR. HAE and OM are physically located in another campus of UCPH. Due to a collective oversight and despite all authors having revised the manuscript, neither OM nor HAE were included in the author list in the revised and published manuscript. To correct this mistake, Osman Mirza and Heidi Asschenfeldt Ernst have now been added to the author list. All authors have agreed to this change and the article has been corrected accordingly to add to the author list the missing authors whose contributions were critical for the study. The contributions section has also been corrected to specify each author’s contribution to the study.

Original author list:

Morten Egevang Jørgensen, Deyang Xu, Christoph Crocoll, David Ramírez, Mohammed Saddik Motawia, Carl Erik Olsen, Hussam Hassan Nour-Eldin, Barbara Ann Halkier

Corrected author list:

Morten Egevang Jørgensen, Deyang Xu, Christoph Crocoll, Heidi Asschenfeldt Ernst, David Ramírez, Mohammed Saddik Motawia, Carl Erik Olsen, Osman Mirza, Hussam Hassan Nour-Eldin, Barbara Ann Halkier

Original author contributions:

MEJ, Identified GTR3 as an indole-specific glucosinolate transporter, Designed the study, Performed the phylogenetic analyses, Biophysical characterization, Data analysis and contributed to generating the gtr1 gtr2 gtr3 triple mutant and micrografting, Wrote the paper based on a draft; DX, Generated the gtr1 gtr2 gtr3 triple mutant, Performed plant work including micro-grafting and glucosinolate analyses of transporter mutants and contributed to study design and preparation of the manuscript; CC, CEO, Performed LC-MS analyses; DR, Performed homology modelling; MSM, Synthesized prunasin; HHN-E, Performed and analysed nitrate uptake assays, Advised and contributed to the study design, Wrote the paper based on a draft, All authors discussed the results and commented on the manuscript; BAH, Advised and contributed to the study design, Wrote the paper based on a draft, All authors discussed the results and commented on the manuscript.

Corrected author contributions:

MEJ, Identified GTR3 as an indole-specific glucosinolate transporter, Designed the study, Performed the phylogenetic analyses, Biophysical characterization, Data analysis and contributed to generating the gtr1 gtr2 gtr3 triple mutant and micrografting, Wrote the paper based on a draft; DX, Generated the gtr1 gtr2 gtr3 triple mutant, Performed plant work including micro-grafting and glucosinolate analyses of transporter mutants and contributed to study design and preparation of the manuscript; CC, CEO, Performed LC-MS analyses; HAE, DR, OM Performed multiple sequence alignments, homology modelling; MSM, Synthesized prunasin; HHN-E, Performed and analysed nitrate uptake assays, Advised and contributed to the study design, Wrote the paper based on a draft, All authors discussed the results and commented on the manuscript; BAH, Advised and contributed to the study design, Wrote the paper based on a draft, All authors discussed the results and commented on the manuscript

Full details for the missing authors:

Name: Heidi Asschenfeldt Ernst

Contribution: HAE contributed to the bioinformatical and computational studies

Affiliation: Department of Drug Design and Pharmacology, Faculty of Health and Medical Sciences, University of Copenhagen, Universitetsparken 2, DK-2100, Copenhagen, Denmark

Competing interests: no competing interests declared

Name: Osman Mirza

Contribution: OM Advised and contributed to the bioinformatical and computational studies

Affiliation: Department of Drug Design and Pharmacology, Faculty of Health and Medical Sciences, University of Copenhagen, Universitetsparken 2, DK-2100, Copenhagen, Denmark

Competing interests: no competing interests declared

