# Supplementary material for: Origin and evolution of transporter substrate specificity within the NPF family
Source: eLife. 2017 Mar 3;6:e19466. doi: 10.7554/eLife.19466 (PMC5336358; doi:10.7554/eLife.19466)
Supplement: Figure 4—source data 1. — Glucosinolate content in rosettes of three-week-old hydroponically grown plants determined by LC-MS. Data presented is one of two individual experiments. Data are given as means and standard deviation (SD) for individual glucosinolates (nmoles/mg FW), total short-chained aliphatic glucosinolates (SC), total long-chained aliphatic glucosinolates (LC), total aliphatic glucosinolates (AG) and total indole glucosinolates (IG). Differences were tested by ANOVA followed by Post-hoc Tukey HSD Calculator multiple comparison (3mtp, 3-methylthiobutylglucosinolate; 3msp, 3-methylsulfinylpropylglucosinolate; 4mtb, 4-methylthiobutylglucosinolate; 4msb, 4-methylsulfinylbutylglucosinolate;5msp,5-methylsulfinylpentylglucosinolate;7mth,7-(methylthio)heptylglucosinolate,7msh,7-ethylsulfinylheptylglucosinolate;8mso, 8-methylsulfinyloctylglucosinolate; I3M, indol-3-ylmethylglucosinolate; 4MOI3M, 4-methoxy-indol-3-ylmethylglucosinolate; NMOI3M, n,-methoxyindol-3-ylmethylglucosinolate). [file elife-19466-fig4-data1.docx]

| **Genotype** | **Short-chained aliphatic glucosinolate** | | | | | | **Long-chained aliphatic glucosinolate** | | | | |  | **Indole glucosinolate** | | | |
| --- | --- | --- | --- | --- | --- | --- | --- | --- | --- | --- | --- | --- | --- | --- | --- | --- |
|  | 3mtp | 3msp | 4msb | 4mtb | 5msp | SC | 7msh | 7mth | 8mso | 8mto | LC | AG | I3m | 4MOI3M | nMOI3M | IG |
| Wildtype  n=10 | 0 | 0.1082 | 0.1218 | 0.3891 | 0.0917 | 0.7107 | 0.2502 | 0.1622 | 0.1401 | 0.5845 | 1.1370 | 1.8478 | 0.1795 | 0.1225 | 0.0227 | 0.3247 |
| ±SD | 0 | 0.0441 | 0.1587 | 0.1875 | 0.0355 | 0.3379 | 0.0985 | 0.1362 | 0.0811 | 0.3144 | 0.5100 | 0.7381 | 0.0613 | 0.0445 | 0.0058 | 0.0884 |
| *gtr3*  n=12 | 0.0060 | 0.1523 | 0.1267 | 0.4760 | 0.0865 | 0.8475 | 0.2916 | 0.1543 | 0.1084 | 0.4562 | 1.0105 | 1.8580 | 0.2122 | 0.2184 | 0.0344 | 0.4650 |
| ±SD | 0.0080 | 0.0394 | 0.0529 | 0.1377 | 0.0216 | 0.2240 | 0.0846 | 0.0704 | 0.0357 | 0.1538 | 0.2830 | 0.3901 | 0.0697 | 0.0534 | 0.0086 | 0.1113 |
| *gtr1 gtr2*  n=8 | 0.0163 | 0.1106 | 0.6580 | 0.4990 | 0.0441 | 1.3280 | 0.1184 | 0.1184 | 0.3915 | 0.3223 | 3.4024 | 4.2347 | 5.5627 | 0.2951 | 0.0463 | 0.7490 |
| ±SD | 0.0202 | 0.0374 | 0.6175 | 0.1729 | 0.0210 | 0.6674 | 0.0580 | 0.0580 | 0.2581 | 0.1348 | 1.8090 | 2.1851 | 2.3173 | 0.1051 | 0.0158 | 0.2606 |
| *gtr1 gtr2 gtr3*  n=10 | 0.0280 | 0.0704 | 0.9104 | 0.4676 | 0.0719 | 1.5482 | 0.1811 | 0.1811 | 0.5614 | 0.3107 | 4.1996 | 5.2528 | 6.8010 | 0.5407 | 0.0838 | 1.3887 |
| ±SD | 0.0214 | 0.0161 | 0.5557 | 0.0782 | 0.0140 | 0.6390 | 0.0944 | 0.0944 | 0.1870 | 0.1124 | 1.3631 | 1.7127 | 2.1920 | 0.1245 | 0.0185 | 0.3533 |
