# Supplementary material for: Origin and evolution of transporter substrate specificity within the NPF family
Source: eLife. 2017 Mar 3;6:e19466. doi: 10.7554/eLife.19466 (PMC5336358; doi:10.7554/eLife.19466)
Supplement: Figure 4—source data 2. — Glucosinolate content in roots of three-week-old hydroponically grown plants determined by LC-MS. Data presented is one of two individual experiments. Data are given as means and standard deviation (SD) for individual glucosinolates (nmoles/mg FW), total short-chained aliphatic glucosinolates (SC), total long-chained aliphatic glucosinolates (LC), total aliphatic glucosinolates (AG) and total indole glucosinolates (IG). Differences were tested by ANOVA followed by Post-hoc Tukey HSD Calculator multiple comparison (3mtp, 3-methylthiobutylglucosinolate; 3msp, 3-methylsulfinylpropylglucosinolate; 4mtb, 4-methylthiobutylglucosinolate; 4msb, 4-methylsulfinylbutylglucosinolate;5msp,5-methylsulfinylpentylglucosinolate;7mth,7-(methylthio)heptylglucosinolate,7msh,7-ethylsulfinylheptylglucosinolate;8mso, 8-methylsulfinyloctylglucosinolate; I3M, indol-3-ylmethylglucosinolate; 4MOI3M, 4-methoxy-indol-3-ylmethylglucosinolate; NMOI3M, n,-methoxyindol-3-ylmethylglucosinolate). [file elife-19466-fig4-data2.docx]

| **Genotype** | **Short-chained aliphatic glucosinolate** | | | | | | **Long-chained aliphatic glucosinolate** | | | | |  | **Indole glucosinolate** | | | |
| --- | --- | --- | --- | --- | --- | --- | --- | --- | --- | --- | --- | --- | --- | --- | --- | --- |
|  | 3mtp | 3msp | 4msb | 4mtb | 5msp | SC | 7msh | 7mth | 8mso | 8mto | LC | AG | I3m | 4MOI3M | nMOI3M | IG |
| Wildtype  n=10 | 0 | 0.0169 | 0.0023 | 0.0107 | 0.0000 | 0.0299 | 0.1825 | 0.5085 | 0.6177 | 0.7539 | 2.0626 | 2.0924 | 0.0103 | 1.9310 | 0.2662 | 2.2075 |
| ±SD | 0 | 0.0231 | 0.0051 | 0.0150 | 0.0000 | 0.0174 | 0.0530 | 0.1404 | 0.1313 | 0.2907 | 0.5238 | 0.5235 | 0.0053 | 0.6747 | 0.1356 | 0.7931 |
| *gtr3*  n=12 | 0 | 0.0121 | 0.0036 | 0.0146 | 0.0000 | 0.0303 | 0.3118 | 0.7317 | 1.0823 | 1.6651 | 3.7908 | 3.8211 | 0.0073 | 0.2840 | 0.0433 | 0.3346 |
| ±SD | 0 | 0.0176 | 0.0062 | 0.0138 | 0.0000 | 0.0191 | 0.1898 | 0.3983 | 0.6359 | 1.2919 | 2.4958 | 2.4921 | 0.0047 | 0.1683 | 0.0269 | 0.1928 |
| *gtr1 gtr2*  n=8 | 0 | 0.0176 | 0.0050 | 0.0145 | 0.0000 | 0.0223 | 0.1849 | 0.4235 | 0.6165 | 1.0825 | 2.2701 | 2.2789 | 0.0057 | 0.3756 | 0.0686 | 0.4402 |
| ±SD | 0 | 0.0055 | 0.0013 | 0.0006 | 0.0000 | 0.0070 | 0.1295 | 0.2965 | 0.4758 | 0.7107 | 1.6452 | 1.6591 | 0.0014 | 0.2654 | 0.0586 | 0.3138 |
| *gtr1 gtr2 gtr3*  n=10 | 0 | 0.0000 | 0.0556 | 0.0157 | 0.0000 | 0.0712 | 0.0380 | 0.0280 | 0.0552 | 0.2769 | 0.3981 | 0.4693 | 0.0088 | 0.3831 | 0.0597 | 0.4517 |
| ±SD | 0 | 0.0000 | 0.0408 | 0.0176 | 0.0000 | 0.0475 | 0.0337 | 0.0232 | 0.0362 | 0.1864 | 0.2173 | 0.2604 | 0.0047 | 0.3388 | 0.0515 | 0.3888 |
