# Supplementary material for: Origin and evolution of transporter substrate specificity within the NPF family
Source: eLife. 2017 Mar 3;6:e19466. doi: 10.7554/eLife.19466 (PMC5336358; doi:10.7554/eLife.19466)
Supplement: Figure 4—source data 3. — Glucosinolate content in rosettes of three-week-old micro-grafted plants determined by LC-MS. Data presented is one of two individual experiments. Data are given as means and standard deviation (SD) for individual glucosinolates (nmoles/mg FW), total short-chained aliphatic glucosinolates (SC), total long-chained aliphatic glucosinolates (LC), total aliphatic glucosinolates (AG) and total indole glucosinolates (IG). Differences were tested by ANOVA followed by Post-hoc Tukey HSD Calculator multiple comparison (3mtp, 3-methylthiobutylglucosinolate; 3msp, 3-methylsulfinylpropylglucosinolate; 4mtb, 4-methylthiobutylglucosinolate; 4msb, 4-methylsulfinylbutylglucosinolate;5msp,5-methylsulfinylpentylglucosinolate;7mth,7-(methylthio)heptylglucosinolate,7msh,7-ethylsulfinylheptylglucosinolate;8mso, 8-methylsulfinyloctylglucosinolate; I3M, indol-3-ylmethylglucosinolate; 4MOI3M, 4-methoxy-indol-3-ylmethylglucosinolate; NMOI3M, n,-methoxyindol-3-ylmethylglucosinolate). [file elife-19466-fig4-data3.docx]

| **Genotype** | **Short-chained aliphatic glucosinolate** | | | | | | **Long-chained aliphatic glucosinolate** | | | | |  | **Indole glucosinolate** | | | |
| --- | --- | --- | --- | --- | --- | --- | --- | --- | --- | --- | --- | --- | --- | --- | --- | --- |
|  | 3mtp | 3msp | 4msb | 4mt | 5msp | SC | 7msh | 7mth | 8mso | 8mto | LC | AG | I3m | 4MOI3M | nMOI3M | IG |
| WT/WT  n=10 | 0.0054 | 0.0238 | 0.1807 | 0.2449 | 0.0221 | 0.4768 | 0.0092 | 0.0026 | 0.0263 | 0.0025 | 0.0405 | 0.5173 | 0.0357 | 0.0176 | 0.0996 | 0.1520 |
| ±SD | 0.0043 | 0.0097 | 0.0613 | 0.118 | 0.0058 | 0.1836 | 0.0050 | 0.0009 | 0.0157 | 0.0011 | 0.0215 | 0.2050 | 0.0114 | 0.0036 | 0.0326 | 0.053 |
| tKO/tKO  n=10 | 0.0351 | 0.0063 | 0.2229 | 0.2606 | 0.0298 | 0.5548 | 0.0251 | 0.0065 | 0.1045 | 0.0109 | 0.1469 | 0.7017 | 0.1524 | 0.0524 | 0.3447 | 0.5606 |
| ±SD | 0.0045 | 0.0035 | 0.0382 | 0.0752 | 0.0056 | 0.0935 | 0.0048 | 0.0021 | 0.0363 | 0.0051 | 0.0469 | 0.1404 | 0.0357 | 0.0239 | 0.1678 | 0.213 |
| qKO/qKO  n=8 | n.d. | n.d. | n.d. | n.d. | n.d. | n.d. | n.d. | n.d. | n.d. | n.d. | n.d. | n.d. | n.d. | n.d. | n.d. | n.d. |
| ±SD |  |  |  |  |  |  |  |  |  |  |  |  |  |  |  |  |
| WT/qKO  n=10 | 0.0090 | 0.0232 | 0.1488 | 0.3017 | 0.0188 | 0.5017 | 0.0068 | 0.0024 | 0.0199 | 0.0030 | 0.0321 | 0.5337 | 0.0379 | 0.0127 | 0.0721 | 0.1265 |
| ±SD | 0.0049 | 0.0099 | 0.0548 | 0.1341 | 0.0058 | 0.199 | 0.0022 | 0.0012 | 0.0080 | 0.0009 | 0.0111 | 0.2101 | 0.0105 | 0.0025 | 0.0219 | 0.028 |
| tKO/qKO  n=16 | 0.0038 | 0.0209 | 0.1617 | 0.1136 | 0.0244 | 0.3245 | 0.0120 | 0.0010 | 0.0301 | 0.0012 | 0.0444 | 0.3689 | 0.2951 | 0.0132 | 0.0518 | 0.8379 |
| ±SD | 0.0026 | 0.0032 | 0.0340 | 0.0714 | 0.0037 | 0.0977 | 0.0020 | 0.0005 | 0.0071 | 0.0006 | 0.0094 | 0.1071 | 0.0540 | 0.0065 | 0.0158 | 0.1537 |
| qKO/WT  n=10 | n.d. | 0.0001 | 0.0015 | 0.0017 | 0.0010 | 0.0043 | 0.0028 | 0.0003 | 0.0083 | 0.0005 | 0.0119 | 0.0162 | n.d. | 0.0029 | 0.0185 | 0.0214 |
| ±SD |  | 0.0002 | 0.0017 | 0.0011 | 0.0004 | 0.0034 | 0.0006 | 0.0004 | 0.0019 | 0.0007 | 0.0036 | 0.0071 |  | 0.0024 | 0.0154 | 0.018 |
| qKO/tKO  n=16 | n.d. | 0.0002 | 0.0032 | 0.0016 | 0.0013 | 0.0063 | 0.0076 | 0.0004 | 0.0373 | 0.0010 | 0.0462 | 0.0526 | 0.2012 | 0.0669 | 0.4378 | 0.7439 |
| ±SD |  | 0.0003 | 0.0026 | 0.0011 | 0.0009 | 0.0048 | 0.0033 | 0.0003 | 0.0195 | 0.0011 | 0.0241 | 0.0289 | 0.0953 | 0.0164 | 0.1184 | 0.195 |
