# Supplementary material for: Origin and evolution of transporter substrate specificity within the NPF family
Source: eLife. 2017 Mar 3;6:e19466. doi: 10.7554/eLife.19466 (PMC5336358; doi:10.7554/eLife.19466)
Supplement: Figure 4—source data 4. — Glucosinolate content in roots of three-week-old micro-grafted plants determined by LC-MS. Data presented is one of two individual experiments. Data are given as means and standard deviation (SD) for individual glucosinolates (nmoles/mg FW). total short-chained aliphatic glucosinolates (SC). Total long-chained aliphatic glucosinolates (LC). total aliphatic glucosinolates (AG) and total indole glucosinolates (IG). Differences were tested by ANOVA followed by Post-hoc Tukey HSD Calculator multiple comparison (3mtp. 3-methylthiobutylglucosinolate; 3msp. 3-methylsulfinylpropylglucosinolate; 4mtb. 4-methylthiobutylglucosinolate; 4msb. 4-methylsulfinylbutylglucosinolate;5msp.5-methylsulfinylpentylglucosinolate;7mth.7-(methylthio)heptylglucosinolate.7msh.7-ethylsulfinylheptylglucosinolate;8mso. 8-methylsulfinyloctylglucosinolate; I3M. indol-3-ylmethylglucosinolate; 4MOI3M. 4-methoxy-indol-3-ylmethylglucosinolate; NMOI3M. n.-methoxyindol-3-ylmethylglucosinolate). [file elife-19466-fig4-data4.docx]

| **Genotype** | **Short-chained aliphatic glucosinolate** | | | | | **Long-chained aliphatic glucosinolate** | | | | |  | **Indole glucosinolate** | | | |
| --- | --- | --- | --- | --- | --- | --- | --- | --- | --- | --- | --- | --- | --- | --- | --- |
|  | 3msp | 4msb | 4mtb | 5msp | SC | 7msh | 7mth | 8mso | 8mto | LC | AG | I3m | 4MOI3M | nMOI3M | IG |
| WT/WT  n=10 | 0.0003 | 0.0109 | 0.0064 | 0.0051 | 0.0227 | 0.0551 | 0.0468 | 0.0996 | 0.1449 | 0.3465 | 0.3692 | 0.0149 | 0.2115 | 0.8424 | 1.0688 |
| ±SD | 0.0005 | 0.0095 | 0.0045 | 0.0049 | 0.0183 | 0.0369 | 0.0194 | 0.0277 | 0.0524 | 0.0928 | 0.1111 | 0.0085 | 0.1967 | 0.1637 | 0.3689 |
| tKO/tKO  n=10 | 0.0011 | 0.0104 | 0.0004 | n.d. | 0.0119 | 0.0176 | 0.0014 | 0.0074 | 0.0081 | 0.0345 | 0.0465 | 0.0079 | 0.0417 | 0.2580 | 0.3076 |
| ±SD | 0.0027 | 0.0231 | 0.0009 |  | 0.0257 | 0.0053 | 0.0035 | 0.0078 | 0.0153 | 0.0265 | 0.0521 | 0.0027 | 0.0145 | 0.0772 | 0.0944 |
| qKO/qKO  n=8 | n.d. | n.d. | n.d. | n.d. | n.d. | n.d. | n.d. | n.d. | n.d. | n.d. | n.d. | n.d. | n.d. | n.d. | n.d. |
| ±SD |  |  |  |  |  |  |  |  |  |  |  |  |  |  |  |
| WT/qKO  n=12 | 0.0062 | 0.0358 | 0.0119 | 0.0053 | 0.0591 | 0.0074 | 0.0103 | 0.0274 | 0.0276 | 0.0728 | 0.1319 | 0.0018 | 0.0066 | 0.0460 | 0.0544 |
| ±SD | 0.0024 | 0.0133 | 0.0054 | 0.0011 | 0.0207 | 0.0013 | 0.0022 | 0.0057 | 0.0015 | 0.0062 | 0.0269 | 4.48E-04 | 0.0018 | 0.0093 | 0.0115 |
| tKO/qKO  n=16 | n.d. | 0.0010 | 0.0007 | n.d. | 0.0017 | 0.0011 | n.d. | 0.0035 | 0.0029 | 0.0074 | 0.0091 | 0.0018 | 0.0015 | 0.0026 | 0.0059 |
| ±SD |  | 0.0012 | 0.0013 |  | 0.0024 | 0.0013 |  | 0.0026 | 0.0025 | 0.0058 | 0.0081 | 0.0027 | 0.0008 | 0.0004 | 0.0039 |
| qKO/WT  n=10 | n.d. | n.d. | n.d. | n.d. | n.d. | 0.0217 | 0.0189 | 0.0476 | 0.0876 | 0.1758 | 0.1758 | 0.0081 | 0.0475 | 0.3134 | 0.369 |
| ±SD |  |  |  |  |  | 0.0005 | 0.0042 | 0.0093 | 0.0179 | 0.0318 | 0.0318 | 0.0017 | 0.0021 | 0.0165 | 0.0203 |
| qKO/tKO  n=16 | n.d. | n.d. | n.d. | n.d. | n.d. | 0.0083 | 0.0015 | 0.0044 | 0.0052 | 0.0195 | 0.0195 | 0.0166 | 0.0566 | 0.5537 | 0.6269 |
| ±SD |  |  |  |  |  | 0.0043 | 0.0007 | 0.0026 | 0.0026 | 0.0020 | 0.0020 | 0.0113 | 0.0358 | 0.2172 | 0.2643 |
